# Correlation between Jejunal Microbial Diversity and Muscle Fatty Acids Deposition in Broilers Reared at Different Ambient Temperatures

**DOI:** 10.1038/s41598-019-47323-0

**Published:** 2019-07-30

**Authors:** Xing Li, Zhenhui Cao, Yuting Yang, Liang Chen, Jianping Liu, Qiuye Lin, Yingying Qiao, Zhiyong Zhao, Qingcong An, Chunyong Zhang, Qihua Li, Qiaoping Ji, Hongfu Zhang, Hongbin Pan

**Affiliations:** 1 Yunnan Provincial Key Laboratory of Animal Nutrition and Feed Science, Faculty of Animal Science and Technology, Yunnan Agricultural University, Kunming, 650201 China; 2 State Key Laboratory of Animal Nutrition, Institute of Animal Science, Chinese Academy of Agricultural Sciences, Beijing, 100193 China; 3 Jiangsu Key Laboratory for Molecular and Medical Biotechnology, College of Life Sciences, Nanjing Normal University, Nanjing, 210023 China; 4 College of Food Science and Technology, Yunnan Agricultural University, Kunming, 650201 China; 5 Yunnan Animal Science and Veterinary Institute, Kunming, 650201 China; 6 Yunnan Mudao Biology Technology Co., Ltd., Kunming, 650201 China

**Keywords:** Metagenomics, Microbiology techniques

## Abstract

Temperature, which is an important environmental factor in broiler farming, can significantly influence the deposition of fatty acids in muscle. 300 one-day-old broiler chicks were randomly divided into three groups and reared at high, medium and low temperatures (HJ, MJ and LJ), respectively. Breast muscle and jejunal chyme samples were collected and subjected to analyses of fatty acid composition and 16S rRNA gene sequencing. Through spearman’s rank correlation coefficient, the data were used to characterize the correlation between jejunal microbial diversity and muscle fatty acid deposition in the broilers. The results showed that *Achromobacter*, *Stenotrophomonas*, *Pandoraea*, *Brevundimonas*, *Petrobacter* and *Variovorax* were significantly enriched in the MJ group, and all of them were positively correlated with the fatty acid profiling of muscle and multiple lipid metabolism signaling pathways. *Lactobacillus* was significantly enriched in the HJ group and exhibited a positive correlation with fatty acid deposition. *Pyramidobacter*, *Dialister*, *Bacteroides* and *Selenomonas* were significantly enriched in the LJ group and displayed negative correlation with fatty acid deposition. Taken together, this study demonstrated that the jejunal microflora manifested considerable changes at high and low ambient temperatures and that jejunal microbiota changes were correlated with fatty acid deposition of muscle in broilers.

## Introduction

Poultry are important protein sources in human diet and hence they are of enormous economic value. The close symbiotic relationship between the host and gut microbiota is crucial in sustaining host health^1^. The gut microbiota help benefit host health through participating in nutrients and energy metabolism, regulating host well-being, and promoting innate and acquired immunity^2–6^. For example, germ-free (GF) C57BL/6J mice consuming a diet high in fat had elevated fecal lipid levels compared with conventional mice^7^. Conventionalized GF zebra fish displayed greater uptake of long- and short-chain fatty acid in the intestinal epithelium under fasted and fed conditions^8^. In addition, decreased lymphatic lipids level was ameliorated in antibiotic-treated rats after fat challenge^9^. A recent study addressed the role of the jejunal microbiome in regulating dietary fat digestion and absorption in a mouse model^10^. A critical role for this microbial community in transducing dietary signals to the host was illustrated to adapt its digestive and absorptive capacity to the dietary fat availability. These above findings demonstrated that the microbiome in jejunum may be a highly relevant target in our efforts to solve the problems associated with fat deposition.

Environment and management related stresses become more severe under intensive poultry farming conditions where selection of birds with higher production performance and better feed conversion efficiency makes them more vulnerable to heat stress and necessitates careful management and preventive steps to decrease possible loss^11,12^. Significant shifts in proportions of fecal microbial population were seen on mice when maintained under the cold environment with higher ratio of Firmicutes/Bacteroidetes^13^. These studies suggest that changes in gut microbiome diversity and/or richness at varied environment temperatures have shown to be associated with fat acid deposition. However, little is known about underlying mechanism that gut microbiota regulate the fatty acid metabolism in broilers at different environmental temperatures. Thus, this study aims to explore the links of microbial diversity and fatty acid composition in broilers raised at different ambient temperatures, thereby providing experimental evidence to understand on how environmental temperatures affect the health and growth performance of broilers via modulation of intestinal microbiota.

## Results

### Fatty acid deposition in breast muscle

As shown in Table 1, the contents of polyunsaturated fatty acid (PUFAs), n-3 PUFAs, n-6 PUFAs, C18:0, C18:2n6c, C18:3n6, C20:2, C20:3n6, C20:3n3 and C22:6n3 in breast muscle of the MJ group were significantly higher (*P* < 0.05) than those in both HJ and LJ groups. Lower monounsaturated fatty acids (MUFA), C16:1, C16:1, C18:ln9c, C20:1 and C18:3n3 of breast muscle in LJ group were observed (*P* < 0.05) compared with the MJ group. As such, both high and low temperature treatments affected fatty acid composition in breast muscle.

**Table 1:** Effects of High and Low Ambient Temperatures on Fatty Acids in Broilers Muscle.

Item	HJ	MJ	LJ	*P*-value
C14:0 (C_14_H_28_O_2_)	1.79 ± 0.90^AB^	2.43 ± 1.29^A^	1.46 ± 1.08^B^	0.018
C14:1 (C_14_H_26_O_2_)	0.58 ± 0.30	0.63 ± 0.38	0.13 ± 0.13	0.084
C15:0 (C_15_H_30_O_2_)	0.27 ± 0.24	0.48 ± 0.26	0.16 ± 0.17	0.766
C16:0 (C_16_H_32_O_2_)	67.03 ± 24.62^AB^	127.37 ± 72.59^A^	54.63 ± 18.58^B^	0.011
C16:1 (C_16_H_30_O_2_)	19.83 ± 12.70^A^	25.19 ± 17.89^A^	7.78 ± 3.74^B^	0.002
C17:0 (C_17_H_34_O_2_)	0.09 ± 0.21^b^	0.74 ± 0.42^ab^	0.27 ± 0.28^a^	0.021
C18:0 (C_18_H_36_O_2_)	26.17 ± 12.15^b^	45.10 ± 22.64^a^	29.86 ± 15.09^b^	0.019
C18:ln9c (C_18_H_34_O_2_)	101.32 ± 66.10^ab^	149.39 ± 100.93^a^	64.16 ± 23.32^b^	0.059
C18:2n6c (C_18_H_32_O_2_)	48.13 ± 15.90^b^	96.86 ± 55.11^a^	46.74 ± 17.96^b^	0.02
C20:0 (C_20_H_40_O_2_)	0.21 ± 0.28	0.40 ± 0.35	0.26 ± 0.22	0.057
C18:3n6 (C_18_H_30_O_2_)	0.83 ± 0.65^b^	1.20 ± 0.62^a^	0.44 ± 0.39^b^	0.033
C20:1 (C_20_H_38_O_2_)	2.74 ± 1.72^AB^	3.73 ± 2.36^A^	1.53 ± 0.61^B^	0.01
C18:3n3 (C_18_H_30_O_2_)	1.04 ± 0.41^ab^	1.55 ± 0.85^a^	0.85 ± 0.23^b^	0.047
C20:2 (C_20_H_36_O_2_)	1.45 ± 0.53^Bb^	2.42 ± 1.03^Aa^	1.08 ± 0.34^Bc^	<0.001
C20:3n6 (C_20_H_34_O_2_)	3.63 ± 0.99^B^	5.93 ± 1.99^A^	2.85 ± 0.78^B^	<0.001
C20:3n3 (C_20_H_36_O_6_)	10.32 ± 2.48^B^	22.73 ± 8.91^A^	10.42 ± 2.50^B^	<0.001
C22:6n3 (C_22_H_32_O_2_)	1.21 ± 1.18^B^	3.55 ± 1.46^A^	2.10 ± 0.68^B^	0.006
Total SFA	114.92 ± 63.91	176.54 ± 96.77	105.64 ± 70.82	0.17
Total PUFA	64.35 ± 20.38^B^	134.86 ± 67.68^A^	64.11 ± 23.03^B^	0.003
Total MUFA	125.28 ± 83.03^ab^	178.82 ± 121.67^a^	73.62 ± 27.32^b^	0.044
PUFA:SFA ratio	0.71 ± 0.03	0.79 ± 0.05	0.76 ± 0.10	0.056
n-3 PUFA	12.80 ± 4.24^B^	27.92 ± 11.10^A^	13.45 ± 3.91^B^	<0.001
n-6 PUFA	52.03 ± 17.03^B^	103.99 ± 57.60^A^	49.83 ± 18.72^B^	0.003
n-6:n-3 ratio	4.70 ± 0.80	3.58 ± 1.00	4.67 ± 2.01	0.14

^A,B^Means in the same row with different superscripts are significantly different. (*P* < 0.01); ^a,b^Means in the same row with different superscripts are significantly different. (*P* < 0.05); n = 8 per treatment group; SFA - saturated fatty acid, PUFA - polyunsaturated fatty acid, MUFA - monounsaturated fatty acid.

### Analysis of microbial diversity

**Figure 1:**
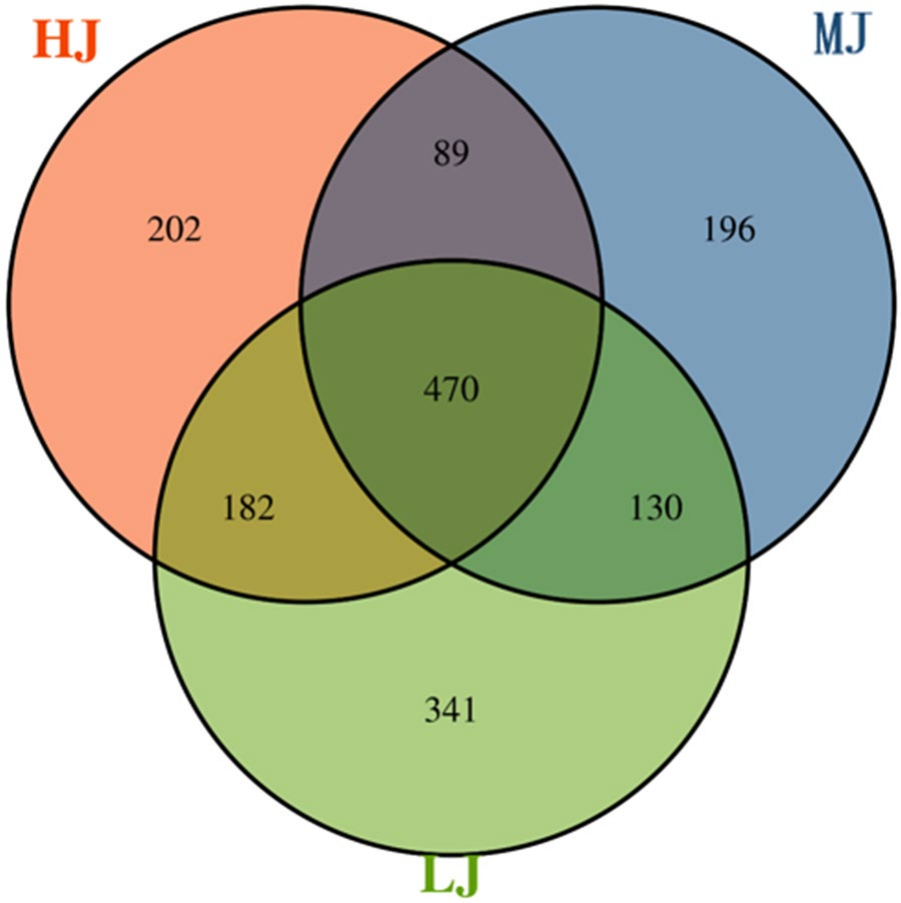
Venn diagram of OTUs clustered at 97% sequence identity across HJ, MJ and LJ groups.

**Figure 2:**
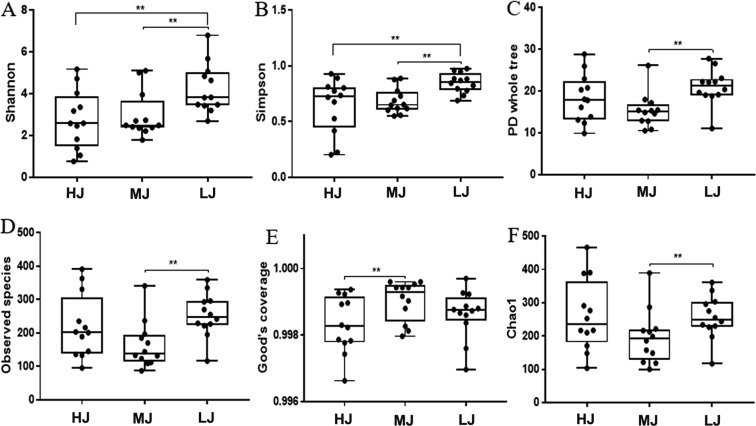
Diversity estimation of the 16 S rRNA gene libraries of the chicken jejunum.(**A**): Shannon index; (**B**):Simpson index; (**C**): PD whole tree index; (**D**): observed species; (**E**): Goods coverage index; (**F**): Chao1 index.

In total, 1,926,570 clean reads were generated from the 36 jejunal samples, which yielded an average of 53,515 clean reads (35,237–64,441) per sample and an average length of 422 bp clean reads. To minimize the effect of the sequencing depth on the measurement of microbial compositions, the library size was rarefied to 32,296 reads per sample using the QIIME pipeline (Supplementary Table S3). With the sequence similarity of at least 97%, 943, 885 and 1123 OTUs were obtained from HJ, MJ and LJ groups and the proportion of core microbiota in the jejunal contents of broilers was 50%, 53% and 42%, respectively **(**Fig. 1). The diversity indices were calculated based on the OTUs of each library (Supplementary Table S4). As shown in Fig. 2, the index of chao1, observed species and PD whole tree in the LJ group were significantly higher (*P* < 0.05) than those in the MJ group; the LJ group had the highest indices of Shannon and Simpson (*P* < 0.05). In comparison, there were no significant differences in diversity indicators between the HJ group and MJ or LJ group except that the Good’s coverage in the MJ group was significantly higher (*P* < 0.05) than those in the HJ group. Overall, our data showed that the low temperature stress increased jejunal microbial diversity.

### Comparison of jejunal bacterial composition at both phylum and genus levels

**Figure 3:**
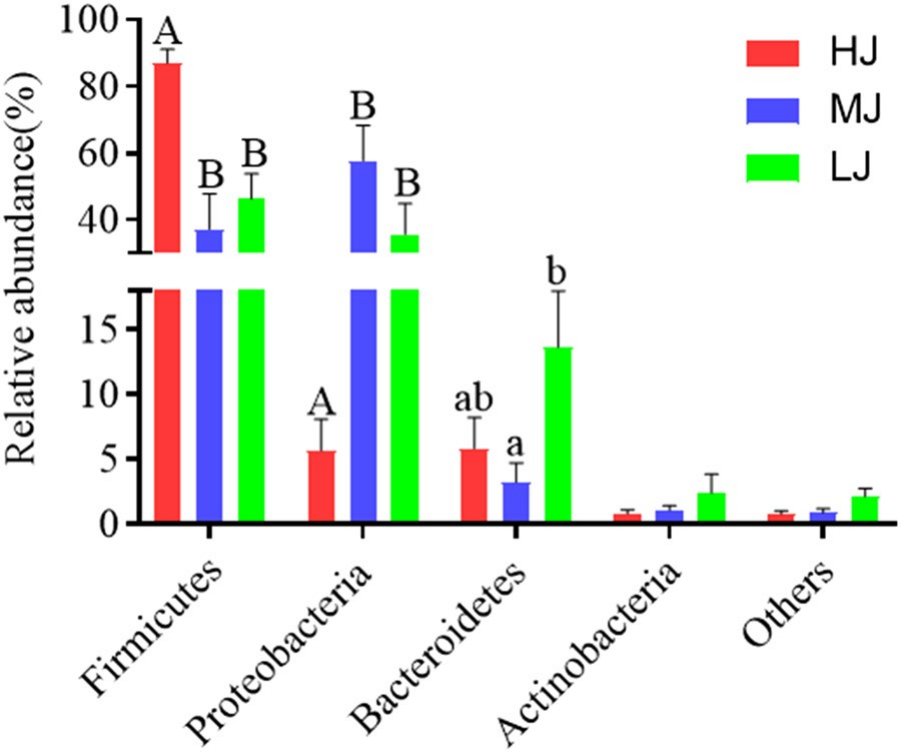
Composition of the dominant microbiome at phylum level (Mean ± MSE). A, B Means in the same row with different superscripts are significantly different. (*P* < 0.01); a, b Means in the same row with different superscripts are significantly different. (*P* < 0.05).

At phylum level, 23 taxa were identified in the LJ group, as were 18 taxa in the HJ group and 17 taxa in the MJ group, respectively (Supplementary Fig. S1). Overall, the jejunal samples from the three groups were all dominated by four phyla, namely Firmicutes, Bacteroidetes, Proteobacteria and Actinobacteria, which together accounted for 99.24%, 99.13% and 97.87% of the total abundance, respectively. The temperate treatments clearly affected the bacterial composition of jejunum. For example, 86.95% of the jejunal bacteria in the HJ group belonged to Firmicutes and the proportion was significantly higher than that of the MJ group (*P* < 0.05). In the low temperature group, Firmicutes was also the most abundant phylum with a proportion of 46.2%, followed by Proteobacteria (35.66%). In contrast, the jejunum of broilers in the MJ group was dominated by Proteobacteria (57.69%), followed by Firmicutes (37.14%). The proportion of Bacteroidetes were significantly different between the LJ and MJ samples (LJ > MJ, 13.63% versus 3.23%, *P* < 0.05). Meanwhile, the ratio of Firmicutes/Bacteroidetes was 14.92, 11.48 or 3.38 in the HJ, MJ or LJ groups, respectively (Fig. 3).

**Figure 4:**
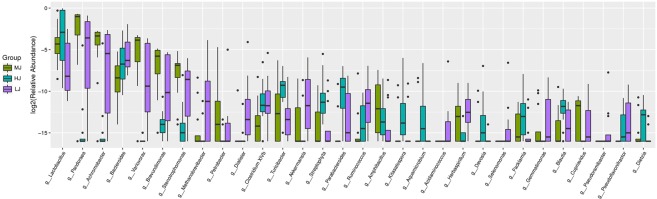
Boxplot of the top 30 most differentially abundant genus in the HJ, MJ and LJ groups.

The genera identified in all jejunal samples were *Lactobacillus*, *Escherichia/Shigella*, *Bacteroides*, *Clostridium XI* and *Faecalibacterium* (Supplementary Fig. S2). The relative abundances of *Achromobacter* (8.69%), *Amphibacillus* (0.12%), *Brevundimonas* (1.71%), *Cupriavidus* (0.02%), *Herbaspirillum* (0.05%), *Pandoraea* (36.89%), *Petrobacter* (0.35%), *Stenotrophomonas* (0.84%) and *Variovorax* (5.31%) were significantly higher in the MJ group than those of the HJ and LJ groups. In comparison with the MJ group, the genera enriched in the HJ were *Aquamicrobium* (0.11%), *Blautia* (0.03%), *Devosia* (0.07%), *Dietzia* (0.02%), *Facklamia* (0.05%), *Kitasatospora* (0.16%), *Lactobacillus* (40.76%), *Parabacteroides* (0.22%), *Streptophyta* (0.28%) and *Turicibacter* (0.19%), whereas those enriched in the LJ group were *Acidaminococcus* (0.10%), *Akkermansia* (0.27%), *Aquabacterium* (0.01%), *Bacteroides* (4.82%), *Brachymonas* (0.005%), *Clostridium XlVb* (0.26%), *Dialister* (0.53%), *Gemmatimonas* (0.04%), *Methanobrevibacter* (0.68%), *Pseudoflavonifractor* (0.028%), *Pseudoramibacter* (0.04%), *Pyramidobacter* (0.004%), *Ruminococcus* (0.15%), *Selenomonas* (0.09%) and *Succinivibrio* (0.01%), respectively (Fig. 4).

### Structures and functions of the broilers jejunal microbiota

**Figure 5:**
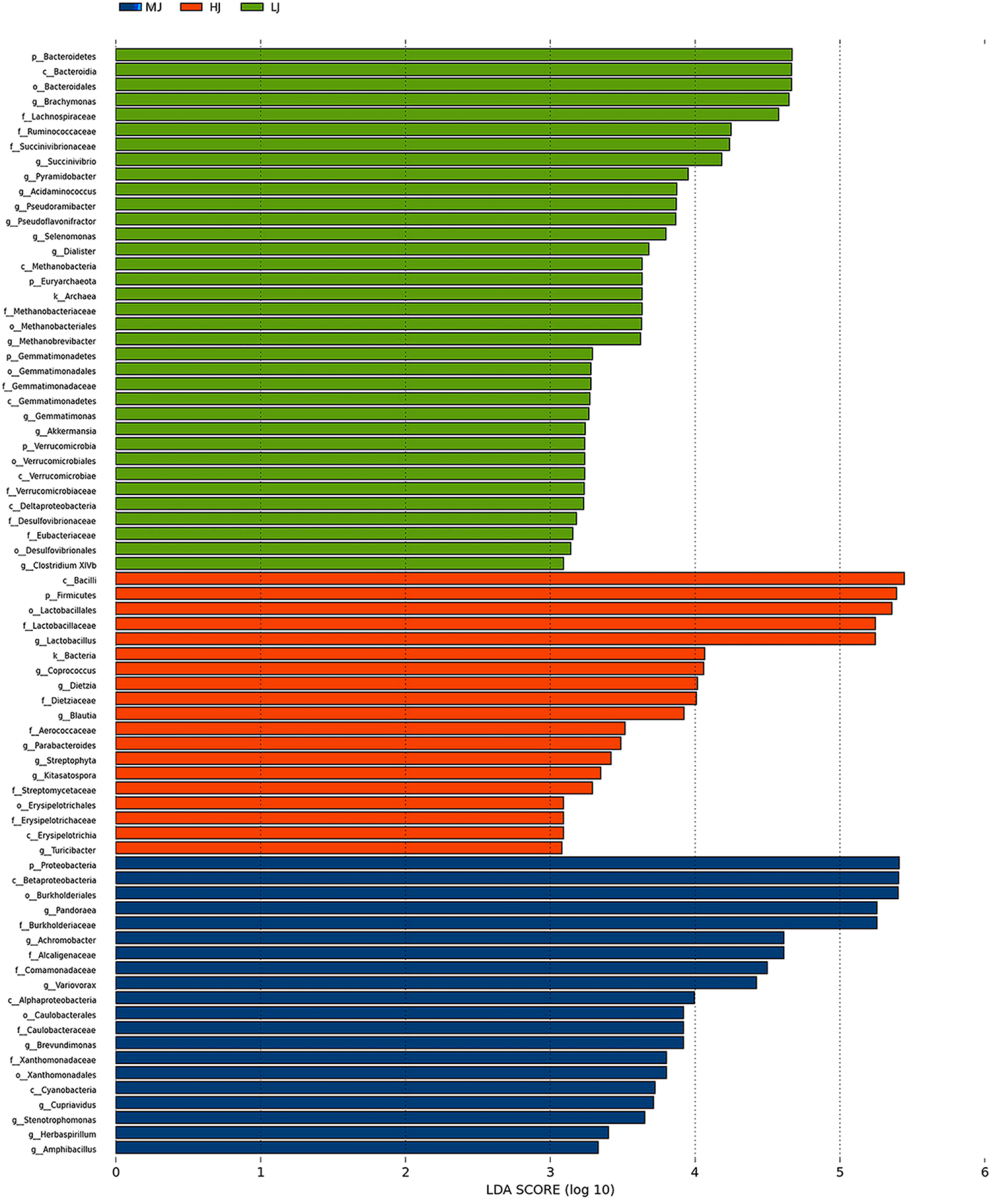
LEfSe identified the most differentially abundant species between HJ (Red), MJ (Blue) and LJ (Green) groups. Only species meeting an LDA significant threshold > 2 are shown.

In order to find the specific bacterial taxa associated with the different temperature treatments, Linear discriminant analysis Effect Size (LefSe) analysis was performed to compare the jejunal microbial compositions in the HJ, MJ and LJ groups. The most differentially abundant phylotypes between the HJ, MJ and LJ groups are shown in Fig. 5. The phylotypes enriched in the MJ group comprised one phylum (Proteobacteria), three classes (Betaproteobacteria, Alphaproteobacteria and Cyanobacteria), three orders (Burkholderiales, Caulobacterales and Xanthomonadales), five families (Burkholderiaceae, Alcaligenaceae, Comamonadaceae, Caulobacteraceae and Xanthomonadaceae) and eight genera (*Pandoraea*, *Achromobacter*, *Variovorax*, *Brevundimonas*, *Cupriavidus*, *Stenotrophomonas*, *Herbaspirillum* and *Amphibacillus*. In addition, the phylotypes enriched in the LJ group included four phyla (Bacteroidetes, Euryarchaeota, Gemmatimonadetes and Verrucomicrobia), five classes (Bacteroidia, Methanobacteria, Gemmatimonadetes, Verrucomicrobiae and Deltaproteobacteria), five orders (Bacteroidales, Methanobacteriales, Gemmatimonadales, Verrucomicrobiales and Desulfovibrionales), eight families (Lachnospiraceae, Ruminococcaceae, Succinivibrionaceae, Methanobacteriaceae, Gemmatimonadaceae, Verrucomicrobiaceae, Desulfovibrionaceae and Eubacteriaceae), nine genera (*Brachymonas*, *Succinivibrio*, *Pyramidobacter*, *Acidaminococcus*, *Pseudoramibacter*, *Pseudoflavonifractor*, *Selenomonas*, *Dialister*, *Methanobrevibacter*, *Gemmatimonas*, *Akkermansia* and *Clostridium XlVb*). Lastly, the phylotypes enriched in the HJ group consisted of one phylum (Firmicutes), two classes (Bacilli and Erysipelotrichia), two orders (Lactobacillales and Erysipelotrichales), five families (Lactobacillaceae, Dietziaceae, Aerococcaceae, Streptomycetaceae and Erysipelotrichaceae), eight genera (*Lactobacillus*, *Coprococcus*, *Dietzia*, *Blautia*, *Parabacteroides*, *Streptophyta*, *Kitasatospora* and *Turicibacter*). The results illustrated the apparent influences of temperature difference on the jejunal microbial composition in broilers.

**Figure 6:**
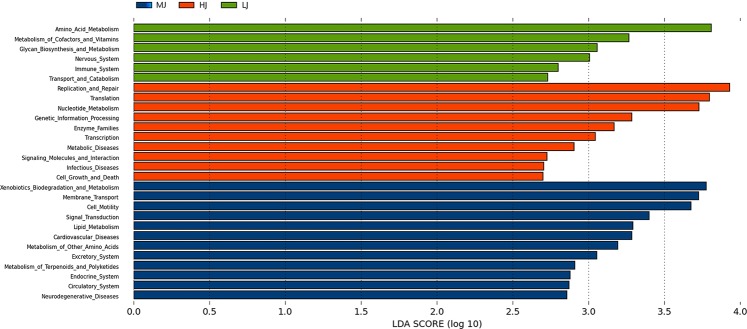
KEGG analysis of enriched signal pathways in the HJ (Red), MJ (Blue) and LJ (Green) groups at L2 hierarchy (LDA > 2).

The functional analysis could shed light on the metabolic differences between the jejunal microbial communities in the HJ, MJ and LJ groups. Based on the PICRUSt-based functional prediction, there were clear differences in the KEGG Orthologs (KO) in the jejunal microbiota between three groups. Overall, twenty-eight signal pathways were significantly different between the HJ, MJ or LJ groups (*P* < 0.05 and LDA > 2) (Fig. 6). Of the three temperature treatments, the medium temperature exhibited the greatest impact on metabolic pathways of jejunal microbiota. The pathways enriched in the MJ group were related to metabolism (e.g., metabolism of terpenoids and polyketides, metabolism of other amino acids, lipid metabolism, xenobiotics biodegradation and metabolism), environmental information processing (e.g., signal transduction, membrane transport), cellular processes (e.g., cell motility), human diseases (e.g., neurodegenerative diseases, cardiovascular diseases), and host physiology (e.g., excretory system, endocrine system, circulatory system). In comparison, ten pathways were enriched in the HJ group, which were associated with environmental information processing (e.g., signaling molecules and interaction), metabolism (e.g., nucleotide metabolism, enzyme families), cellular processes (e.g., cell growth and death), human diseases (e.g., metabolic diseases, infectious diseases), and gene regulation (e.g., genetic information processing, replication and repair, translation, transcription). The low temperature treatment had a relatively low impact on pathways, as six pathways were enriched in the LJ group, which were related to metabolism (e.g., amino acid metabolism, metabolism of cofactors and vitamins, glycan biosynthesis and metabolism), host physiology (e.g., nervous system, immune system), and cellular processes (e.g., transport and catabolism). Hence, the different temperature treatments greatly affected the jejunal microbial functions of the broilers.

### The association of microbial composition, fat acid deposition and metabolic pathways

**Figure 7:**
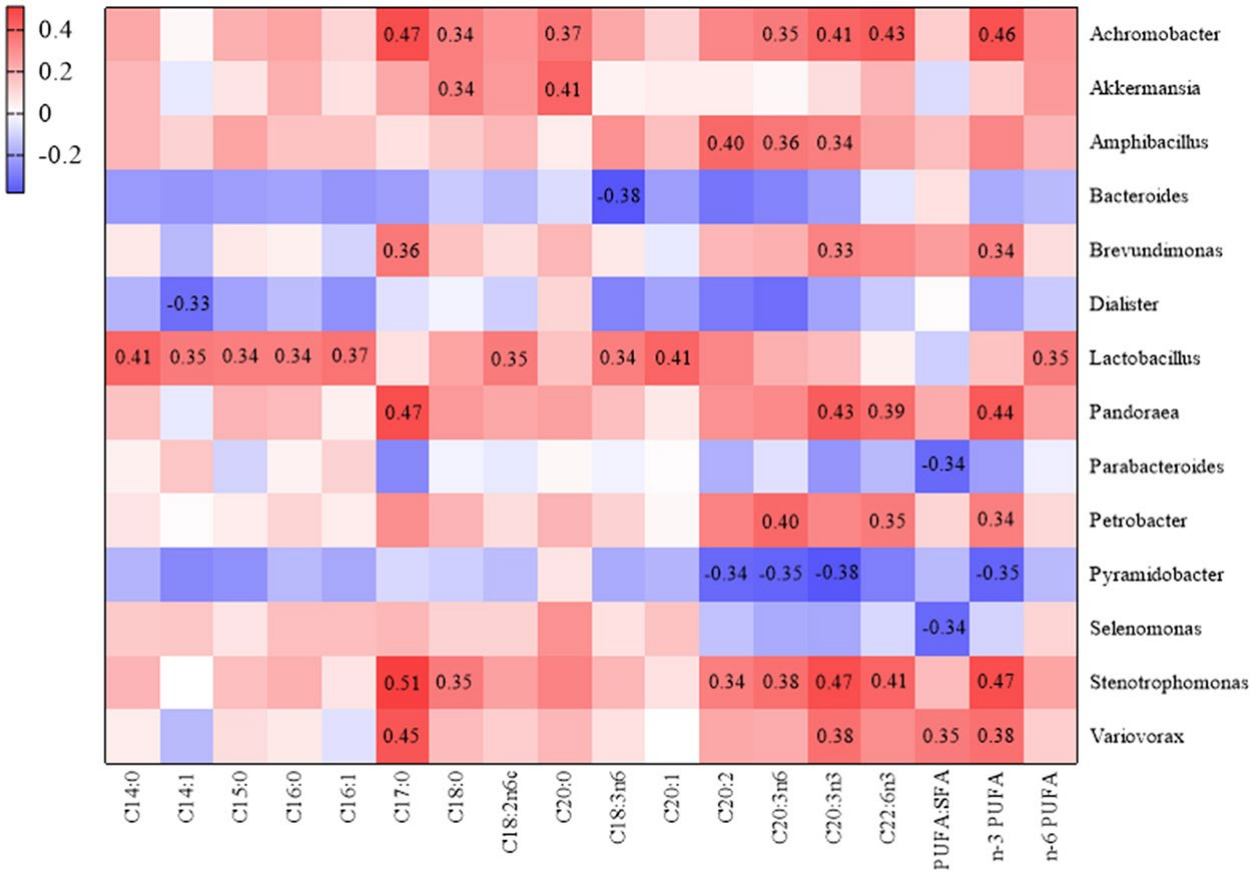
Heatmap analysis of the correlation between microbial changes and fat acid deposition, the values in the figure are Spearman’s correlation coefficient. (*P*-value < 0.05).

To investigate the correlation between alterations in jejunal microbial composition and host fat acid deposition in the broiler reared at different temperatures, a heatmap of dominant genera and fatty acid contents was constructed for the HJ, MJ or LJ groups (Fig. 7). Complex interactions between microbes and fatty acid contents were revealed in the broilers of the MJ group. For example, *Achromobacter* was positively correlated with the contents of C17:0, n-3 PUFA, C22:6n3, C20:3n3, C20:0, C20:3n6 and C18:0; *Amphibacillus* was positively correlated with the contents of C20:2, C20:3n6 and C20:3n3; *Brevundimonas* was positively related with the contents of C17:0, n-3 PUFA and C20:3n3; *Pandoraea* was positively correlated with the contents of C17:0, n-3 PUFA, C20:3n3 and C22:6n3; *Petrobacter* was positively associated with the contents of C20:3n6 C22:6n3 and n-3 PUFA; *Stenotrophomonas* was positively correlated with the contents of C17:0, C20:3n3, n-3 PUFA, C22:6n3, C20:3n6, C18:0 and C20:2; *Variovorax* was positively related with the contents of C17:0, n-3 PUFA, C20:3n3 and ratio of PUFA:SFA. *Lactobacillu*s in the broiler jejunum of HJ group was positively correlated with the contents of C20:1, C14:0, C16:1, C18:2n6c, n-6 PUFA, C14:1, C18:3n6, C15:0 and C16:0, whereas *Parabacteroides* was negatively correlated with the ratio of PUFA: SFA in the breast muscle. In the broilers maintained at the low temperature, the correlations between bacteria in the jejunum and fatty acid contents in breast muscle were very different from that of the MJ and HJ groups: *Pyramidobacter* was negatively correlated with the contents of C20:3n3, C20:3n6, n-3 PUFA and C20:2; *Dialister* was negatively correlated with the contents of C14:1; *Bacteroides* was negatively with the contents of C18:3n3; *Selenomonas* was negatively with the ratio of PUFA:SFA; *Akkermansia* was positively correlated with the contents of C18:0 and C20:0. Hence, the high and low temperature treatments clearly affected the correlations between the jejunal microbiota and muscle fatty acid contents.

**Figure 8:**
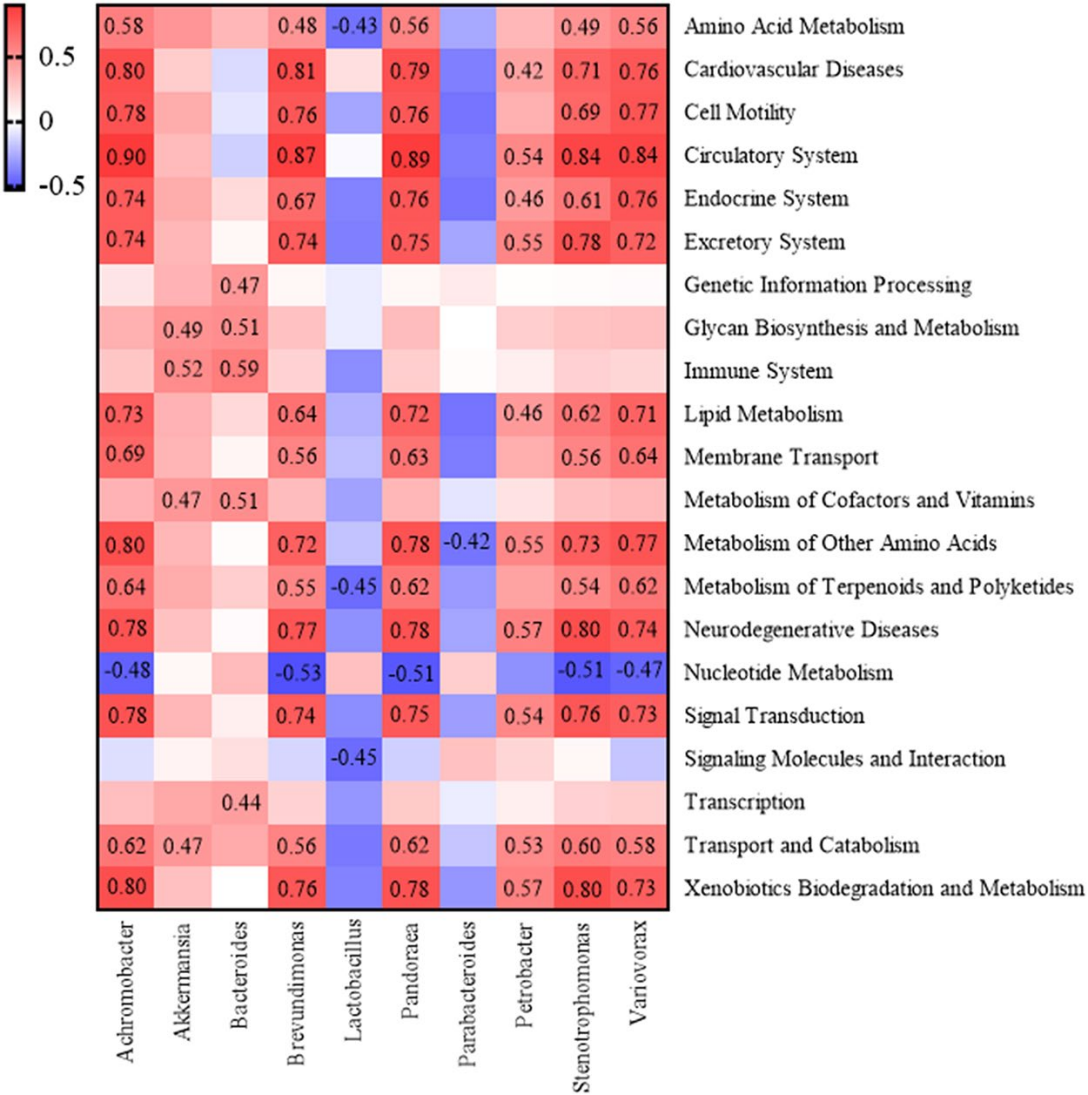
Heatmap analysis of the correlation between microbial changes and pathways, the values in the figure are Spearman’s correlation coefficient. (*P*-value < 0.01).

To further examine the underlying mechanisms whereby jejunal genera were implicated in fatty acid deposition in the muscle, the dominant genera associated with fatty acid deposition were first selected and the pathways enriched at L2 level were identified (Fig. 8). In the MJ group, there were positive correlations between genera of *Achromobacter*, *Stenotrophomonas*, *Pandoraea*, *Brevundimonas*, *Petrobacter* and *Variovorax* and multiple pathways such as circulatory system, cardiovascular diseases, xenobiotics biodegradation and metabolism, metabolism of other amino acids, neurodegenerative diseases, signal transduction, lipid metabolism, transport and catabolism, endocrine system and excretory system signaling pathways. In the HJ group, *Lactobacillus* was negatively correlated with metabolism of terpenoids and polyketides, amino acid metabolism and signaling molecules and interaction signaling pathways. In the LJ group, *Akkermansia* and *Bacteroides* were positively correlated with immune system, metabolism of cofactors and vitamins, glycan biosynthesis and metabolism signaling pathways. As such, the complexity of microbe-pathway associations was relatively low in both the HJ and LJ groups in comparison with that of the MJ group.

## Discussion

The proximal small intestine serves as the major region of macronutrient digestion and absorption, whereas jejunum is a structurally and functionally distinct region of the alimentary tract that connects the upper portion of the small intestine (duodenum) to the lower portion of the small intestine (ileum). Complex interactions among dietary cues, gut endocrine responses, bile release, exocrine function of the pancreas, and absorptive enterocyte function are indispensible for efficient absorption of lipids and other dietary nutrients. Studies based on high-throughput sequencing were used to investigate the gut microbial diversity in the small intestine of poultry^14^. For example, it has been reported that the gastrointestinal microbiota in broilers was predominantly occupied by members of Firmicutes, Bacteroidetes, Proteobacteria and Actinobacteria^15–17^. Firmicutes and Proteobacteria, which dominate in the small intestine, have been shown to be more tolerant of environmental influences. Bacteroidetes was resistant to bile but its abundance reduced in the small intestine and increased in the large intestine^18–20^. However, few studies have investigated the effects of ambient temperature on the interaction between fatty acid deposition and gut microbiota in broiler. In this study, we used high-throughput sequencing analysis to examine whether different temperatures affected jejunal microbiota, fatty acids deposition of breast muscle, and their correlations. In comparison with the MJ-treated broilers, those in the HJ group displayed a significant increase in Firmicutes by 49.81% (*P* < 0.01). This is consistent with a previous study that high ambient temperature (31 ± 1 °C) increased Firmicutes abundance^21^. Firmicutes represents the largest group of the gut microbes in mouse and human^22^ and many species in the phylum are able to produce endospores, which are resistant to desiccation and other harsh conditions^23^. Firmicutes has been shown to be involved in energy resorption and is potentially related to the development of diabetes and obesity in human^24–26^. Our results and these previous studies concur to suggest that the significantly increased abundance of Firmicutes could be beneficial for broilers to adapt and grow healthy at high ambient temperature. On the other hand, jejunal Bacteroidetes exhibited significant increase in relative abundance (10.40%) in broilers maintained at the lower temperature compared with that of the MJ group. Taken together, the temperature-dependent dynamics of Firmicutes and Bacteroidetes may help broilers to adapt to the changes of ambient temperature.

Gut Bacteroidetes has been reported to be associated with degradation of high molecular weight organic matter, i.e., proteins and carbohydrates, in human or animals^27^. Members in Bacteriodetes can break down starch and other polymeric substances via producing α-amylase, α-1,2-mannosidase and endo-1,4-β-mannosidase^28^. In addition to digestive function, Bacteroidetes in the gut has also been linked to multiple metabolic functions including nutrient digestion and calories absorption^29^. In addition, intestinal Bacteroidetes may have the potential in ameriolating metabolic syndrome, mood impairment and neurologic disorders, probably through modulating immune responses, affecting nutrients metabolism and regulating the gut-brain-axis^30–32^. In comparison with that of the MJ group, the jejunal Firmicutes/Bacteroidetes ratio in the broilers of HJ group increased and in the LJ group decreased. Enhancement in Firmicutes/Bacteroidetes ratio was shown to be related to fat deposition positively and weight loss negatively in human^33,34^. Members in Firmicutes are involved in butyrate and propionate synthesis, as are microbes of Bacteroidetes in propionate synthesis, thereby facilitating short chain fatty acid metabolism^27^.

For human, chicken meat is one of the main sources of PUFA, especially n-3 PUFA, and its fatty acid composition is modulated by altering lipid intake and absorption levels^35,36^. In addition to n-3 PUFAs, chicken meat also contains other fatty acids including C18:3n-3 (α-LA), C20:5n-3 (EPA) and C22:6n-3 (DHA), which have health benefits in preventing brain, retina and cardiovascular diseases^37–39^. Meanwhile, it has been demonstrated that n-3 PUFA exhibited curative effects on bronchial asthma, neuropsychiatric disorders and cognitive brain function in children and can also prevent future cardiovascular events in adults^40^. In our study, the contents of total PUFAs, n-3 PUFAs, n-6 PUFA, C16:0, C17:0, C18:2n6c, C20:2, C20:3n6, C20:3n3 and C22:6n3 in breast muscle of broilers from the MJ group were significantly higher (*P* < 0.01) than those in the both HJ and LJ groups. Compared with the MJ group, the broilers from the LJ group had lower proportions of C14:1, C15:0, C16:1 and C18:3n6 (*P* < 0.01) as well as total MUFA C18:ln9c, C20:1 and C18:3n3 (*P* < 0.05). Moreover, the contents of C15:0, C18:0 and the ratio of PUFA: SFA in HJ group were significantly lower (*P* < 0.05) than those in the MJ group. These results collectively suggested that both high and low temperature treatments influenced fatty acid deposition in breast muscle. At the genus level, *Achromobacter*, *Amphibacillus*, *Brevundimonas*, *Pandoraea*, *Petrobacter*, *Stenotrophomonas* and *Variovorax* were significantly enriched in MJ group and were positively correlated with the fatty acid deposition. We also observed that *Achromobacter*, *Stenotrophomonas*, *Pandoraea*, *Brevundimonas* and *Variovorax* were positively correlated with pathways involved in lipid metabolism.

Studies in human and animal models have shown that presence of some *Lactobacillus* species alters gut microbiota composition and reduce inflammation in the gut^41–44^. In a recent study, *Lactobacillus johnsonii* mitigated the *Clostridium perfringens*-resulted impact on growth, lipid levels, fatty acid composition and nutritional quality in the broiler’s meat^45^. In particular, the lactobacilli treatment improved the fatty acids profiling in broilers challenged with with *Clostridium perfringens* by increasing the contents of multiple fatty acids, namely C18:3n-3 (α-LA), C20:4n-6, C20:5n-3 (EPA), C22:4n-6, C22:5n-3, C22:6n-3 (DHA), total PUFA and n-3 PUFA and the PUFA: SFA ratio^45^. In our study, jejunal *Lactobacillus* abundance was increased by 26.98% (*P* = 0.029) and 36.77% (*P* = 0.004) in broilers of the HJ group compared with that of the MJ and LJ groups. Furthermore, jejunal *Lactobacillus* was positively correlated with the contents of C20:1, C14:0, C16:1, C18:2n6c, n-6 PUFA, C14:1, C18:3n6, C15:0 and C16:0 in breast muscle, corroborating the previous findings that probiotics supplementation improves fatty acids composition in the meat of broilers^46^.

In the broilers of the LJ group, *Pyramidobacter*, *Dialister*, *Bacteroides*, *Selenomonas* and *Akkermansia* were enriched. Among those, *Pyramidobacter*, *Dialister*, *Bacteroides*, and *Selenomonas* were negatively correlated with the contents of C20:3n3, C20:3n6, n-3 PUFA, C14:1, C18:3n6, C20:2 and ratio of PUFA: SFA, whereas *Akkermansia* was positively correlated with the contents of C18:0 and C20:0. In mice fed with a high fat diet, oral administration of *Akkermansia* improved glucose tolerance and increased the numbers of goblet cells and adipose tissue-resident CD4 Foxp3 regulatory T cells^47^ or boosted intestinal levels of endocannabinoids which can control inflammation, enhance the gut barrier, and induce gut peptide secretion^48^. In our study, the abundances of *Akkermansia* and *Bacteroides* were positively correlated with the signaling pathways related to immune system, glycan biosynthesis and metabolism and metabolism of cofactors and vitamins. *Bacteroides* manifests a high degree of host specificity that reflects the individual differences in the digestive system of the host animals^49^. Further study is needed to elucidate the underlying mechanisms of *Akkermansia* and *Bacteroides* that contribute to the improvement in the immune and health of broilers at low ambient temperature.

In summary, we analyzed the fatty acid composition of breast muscle in broilers reared under different ambient temperatures. Our analysis revealed that the content of all tested muscle fatty acids on broiler decreased in either HJ or LJ group when compared with MJ group, with the LJ group exhibiting an overall greater reduction. Next we analyzed the differences in the phylogenetic and functional metagenomic overall structure of broiler jejunum microbiota in all three groups. In comparison with the MJ group, the HJ and LJ groups exhibited many changes: the diversity of jejunum microbiota increased; the relative abundances of several bacteria related to intestinal homeostasis (especially *Lactobacillus*, *Akkermansia* or *Bacteroides*) altered; large numbers of biochemical pathways associated with environmental, cellular processes and metabolic balance were enriched. Furthermore, we investigated the association between changes in jejunal microbiota composition and fatty acids profiling in muscle at different tempratures. We found that most enriched genera in the jejunum of MJ-treated broilers were positively correlated with fatty acids deposition and lipid metabolism pathway, although many enriched genera in the LJ group have undesirable impact on fat acid deposition. *Lactobacillus* showed the strongest relevance to fatty acids deposition in the HJ group, whereas *Akkermansia* and *Bacteroides* were positively correlated with immune system pathways. However, our study is inadequate to draw a concrete conclusion on how the jejunal microbial community regulates fatty acid deposition and host health under different ambient temperatures. Nevertheless, this study provides an important insight to the possible role of jejunal microbes on the regulation of fatty acids deposition and the adaption to temperature changes.

## Materials and Methods

### Animal ethics statement

All experiments performed in this study were approved by the International Animal Care and Use Committee of the Yunnan Agricultural University (permission code: YAUACUC01; date of publication: 10 July 2013). The study complied with the guidelines of the institutional administrative committee and ethics committee of laboratory animals.

### Experimental design and animal management

A total of 300 one-day-old chicks were purchased from a local supplier (Hunan Shuncheng Industrial Co., Ltd. Hunan, China). The birds were randomly divided into three groups, namely the high temperature group (HJ), medium temperature group (MJ), and low temperature group (LJ), respectively. The housing temperature was designed as a parallel, decremental process such that it started at 36.5 °C (HJ), 33.5 °C (MJ) or 30.5 °C (LJ) on the first day and was decreased by 0.5 °C or 0 °C per day until it reached a temperature of 22 °C, 19 °C or 16 °C on d42, respectively (Supplementary Table S1). Each group contained 100 birds. Except for the temperature difference, chickens in all three groups received same treatments (Supplementary Table S2). The broilers were reared in temperature-controlled house covered with rice husk litter (8 cm high), where they were offered with access to feed and water ad libitum. In addition, they were provided with the following light/dark cycles: 24/0 on d1, 23/1-18/6 during d2 to d8, 12/12 during d9 to d21, 18/6 during d22 to d35, and 18/6-23/1 during d36 to d42 (light period increased from 18 h on day 36 to 23 h on day 42); the light intensity was 30-60 lux for chickens with a weight < 160 g (about 1–8 d) and 5–10 lux for broilers with weight > 160 g (about 9–42 d). The relative humidity was 30–50% from d1 to d7, 40–60% from d8 to d21, and 50–70% from d22 to d42. The broilers were provided the access to feed and water ad libitum.

### Sample collection

On day 42, twelve birds from each group were randomly selected and sacrificed by cervical dislocation. Jejunal chyme was removed, preserved in liquid nitrogen, and subsequently used for DNA extraction and PCR amplification, with the both sides of breast major muscle collected and stored at −20 °C for measurement of fatty acid content and composition.

### Determination of fatty acid contents

Fatty acid content was determined according to the previous studies^50,51^. Briefly, breast muscle samples were freeze-dried and ground for extraction and methylation of fatty acids, which was followed by fatty acid analysis using an HP6890 gas chromatograph equipped with a flame-ionization detector and a DB-23 capillary column (0.25 mm × 60 m × 0.25 μm; J&W Scientific, Folsom, CA). The following oven temperature program was used: 180 °C for 10 min, increased to 220 °C at 4 °C/min and held for 15 min, and increased to 250 °C at 3 °C/min. A 1-μL sample was injected with a split ratio of 1:20 at an inlet temperature of 250 °C. Helium was used as the carrier gas at a constant flow rate of 1.1 mL/min. Individual fatty acids were identified by comparison of their retention times with those in the standard mix of fatty acids (Supelco 37 component FAME mix). Contents of individual fatty acids (% of total fatty acids) were determined using a C19:0 internal standard from Sigma (USA).

### DNA extraction and PCR amplification

Genomic DNA was extracted from jejunal samples using the QIAamp ® Fast DNA Stool Mini Kit (Qiagen, Cat No.19593) according to manufacturer’s protocols. The DNA samples were used as the template for PCR. The V3-V4 region of the bacteria 16 S ribosomal RNA genes were amplified using the primers 341 F 5′-CCTACGGGRSGCAGCAG-3′ and 806 R 5′-GGACTACVVGGGTATCTAATC-3′ and the following cycles: a denaturing step at 95 °C for 3 min, followed by 30 cycles at 98 °C for 20 s, 58 °C for 15 s, and 72 °C for 20 s and a final extension at 72 °C for 5 min. PCR reactions were performed in 30 μL mixture containing 15 μL of 2 × KAPA Library Amplification ReadyMix, 1 μL of each primer solution (10 μM), and 50 ng of template DNA.

### Illumina HiSeq PE250 sequencing

Amplicons were electrophoresed in 2% agarose gels, before they were extracted from the gel and purified using the AxyPrep DNA Gel Extraction Kit (Axygen Biosciences, Union City, CA, U.S.) according to the manufacturer’s instructions and quantified using Qubit®2.0 (Invitrogen, CA, U.S.). Prepared library was sequenced on a HiSeq platform (Illumina, Inc., CA, USA) for paired-end reads of 250 bp, which were overlapping on their 3 ends for concatenation into longer contig. DNA extraction, library construction and sequencing were performed at Realbio Genomics Institute (Shanghai, China).

### Processing of sequencing data

Tags, trimmed of barcodes and primers, were subjected to analysis of rest lengths and average base quality. 16S tags were restricted between 220 bp and 500 bp such that the average Phred score of bases was higher than 20 (Q20) and there were not more than 3 ambiguous N. The copy number of tags was enumerated and redundant tags were removed. Only the tags with frequency higher than 1 were clustered into operational Taxonomic Units OTUs, each of which had a representative tag^52^. OTUs were clustered with 97% similarity using UPARSE (http://drive5.com/uparse/) and chimeric sequences were identified and removed using Userach (version 7.0). Each representative tag was assigned to a taxon by RDP Classifier against the RDP database (http://rdp.cme.msu.edu/) using confidence threshold of 0.8 OTU profiling table. Alpha/beta diversity was analyzed by python scripts of Qiime. According to the abundance of OTUs, the total and unique OTUs in each sample or group are calculated. Venn diagram was used to determine the number of specific OTUs in each group^53^. According to the bacterial annotation, the relative abundance of sequences of each sample annotated to the taxonomic levels (Kingdom, Phylum, Class, Order, Family, Genus) was counted, and the OTU abundance table was obtained according to the number of sequences in each OUT^54,55^. Alpha diversity of species diversity in each individual sample, including chao1 values, Good’s Coverage values, Shannon indices, observed species indices, PD whole tree indices, and Simpson indices^56,57^, were calculated using QIIME software based on the OTU results to generate the corresponding dilution curve^58^. QIIME software and iterative algorithm were used to calculate the difference between the OTUs classification abundance information^59^.

### Statistical analysis

Experimental data including fatty acid and microbiota abundances were analyzed using the SPSS 22.0 software (IBM SPSS Statistics for Windows; NY: IBM Corp). Shapiro-Wilk test was applied to assess normality. After logarithmic transformation, only fatty acid and part of the phylum level microbial data (including Bacteroidetes, Actinobacteria, Fusobacteria) displayed normal distribution. The General linear model analysis with Duncan multiple comparison test were used for parametric data and Kruskal–Wallis ANOVA performed on ranks were used for the other phylun level microbiota and genus level microbiota. The fatty acid data are expressed as the mean± standard error of mean (SEM).For Firmicutes/Bacteroidetes ratios, calculations were performed for each group using the average relative abundance of the two phyla.

Linear discriminant analysis (LDA) effect size (LEfSe) method was used to identify the most differentially abundant OTUs between the three groups^60^. Phylogenetic Investigation of Communities by Reconstruction of Unobserved States (PICRUSt) based on closed-reference operational taxonomic unit (OTU) was used to predict the abundance of functional categories the Kyoto Encyclopedia of Genes and Genomes (KEGG) ortholog (KO)^61,62^.

To assess the correlation between dominant genus, KEGG pathways and fatty acids, we performed the Spearman’s test in GraphPad Prism 7.0^63^. *P* < 0.05 was considered as statistical significance.

## Supplementary information

Supplementary Material

## References

[1] Misurini LMC (2018). Intestinal Microbiota, Obesity and Insulin Resistance—What Are the Relationships?. Health.

[2] Hou Q (2016). Differential fecal microbiota are retained in broiler chicken lines divergently selected for fatness traits. Sci. Rep..

[3] Bäckhed F (2004). The gut microbiota as an environmental factor that regulates fat storage. Proc. Natl. Acad. Sci. USA.

[4] Chou Chieh Jason, Membrez Mathieu, Blancher Florence (2008). Gut Decontamination with Norfloxacin and Ampicillin Enhances Insulin Sensitivity in Mice. Nestlé Nutrition Workshop Series: Pediatric Program.

[5] Koren O (2012). Host remodeling of the gut microbiome and metabolic changes during pregnancy. Cell.

[6] Ridaura VK (2013). Gut microbiota from twins discordant for obesity modulate metabolism in mice. Science.

[7] Rabot S (2010). Germ-free C57BL/6J mice are resistant to high-fat-diet-induced insulin resistance and have altered cholesterol metabolism. FASEB J..

[8] Semova I (2012). Microbiota regulate intestinal absorption and metabolism of fatty acids in the zebrafish. Cell Host Microbe.

[9] Sato H (2016). Antibiotics suppress activation of intestinal mucosal mast cells and reduce dietary lipid absorption in Sprague-Dawley rats. Gastroenterology.

[10] Martinez-Guryn K (2018). Small Intestine Microbiota Regulate Host Digestive and Absorptive Adaptive Responses to Dietary Lipids. Cell Host Microbe.

[11] Lara LJ, Rostagno MH (2013). Impact of heat stress on poultry production. Animals.

[12] Abdelqader A, Al-Fataftah AR (2016). Effect of dietary butyric acid on performance, intestinal morphology, microflora composition and intestinal recovery of heat-stressed broilers. Livestock Science.

[13] Chevalier C (2015). Gut microbiota orchestrates energy homeostasis during cold. Cell.

[14] Zhang C (2018). A Comparison of Homogenization vs. Enzymatic Lysis for Microbiome Profiling in Clinical Endoscopic Biopsy Tissue Samples. Front. Microbiol..

[15] Gong J (2010). 16s rrna gene-based analysis of mucosa-associated bacterial community and phylogeny in the chicken gastrointestinal tracts: from crops to ceca. Fems Microbiol. Ecol..

[16] Corrigan A, Horgan K, Clipson N, Murphy RA (2011). Effect of dietary supplementation with a saccharomyces cerevisiae mannan oligosaccharide on the bacterial community structure of broiler cecal contents. Appl Environ. Microbiol..

[17] Hume ME (2011). Use of pyrosequencing and denaturing gradient gel electrophoresis to examine the effects of probiotics and essential oil blends on digestive microflora in broilers under mixed eimeria infection. Foodborne Pathog. Dis..

[18] Angelakis E (2015). A metagenomic investigation of the duodenal microbiota reveals links with obesity. Plos One.

[19] Donaldson GP, Lee SM, Mazmanian SK (2015). Gut biogeography of the bacterial microbiota. Nat. Rev. Microbiol..

[20] Turnbaugh PJ (2009). The effect of diet on the human gut microbiome: a metagenomic analysis in humanized gnotobiotic mice. Sci. Transl. Med..

[21] Wang XJ (2018). Effects of high ambient temperature on the community structure and composition of ileal microbiome of broilers. Poult. Sci..

[22] Ley RE, Peterson DA, Gordon JI (2006). Ecological and evolutionary forces shaping microbial diversity in the human intestine. Cell.

[23] Filippidou, S. *et al*. A combination of extreme environmental conditions favor the prevalence of endospore-forming firmicutes. *Front*. *Microbiol*. 7 (TBS-0015-2012), 10.3389/fmicb.2016.01707 (2016). 10.3389/fmicb.2016.01707 PMC5094177 27857706

[24] Ley RE, Turnbaugh PJ, Klein S, Gordon JI (2006). Microbial ecology: human gut microbes associated with obesity. Nature.

[25] Ley RE (2005). Obesity alters gut microbial ecology. Proc. Natl. Acad. Sci. USA.

[26] Komaroff AL (2017). The microbiome and risk for obesity and diabetes. JAMA..

[27] Thomas F, Hehemann JH, Rebuffet E, Czjzek M, Michel G (2011). Environmental and Gut Bacteroidetes: The Food Connection. Front. Microbiol..

[28] Polansky O (2016). Important metabolic pathways and biological processes expressed by chicken cecal microbiota. Appl. Environ. Microb..

[29] Gibiino Giulia, Lopetuso Loris Riccardo, Scaldaferri Franco, Rizzatti Gianenrico, Binda Cecilia, Gasbarrini Antonio (2018). Exploring Bacteroidetes: Metabolic key points and immunological tricks of our gut commensals. Digestive and Liver Disease.

[30] Ivanov II (2008). Specific microbiota direct the differentiation of il-17-producing t-helper cells in the mucosa of the small intestine. Cell Host Microbe.

[31] Emm Q (2017). Microbiota-brain-gut axis and neurodegenerative diseases. Curr. Neurol. Neurosci..

[32] Dinan TG, Cryan JF (2016). Gut instincts: microbiota as a key regulator of brain development, ageing and neurodegeneration. J. Physiol..

[33] Turnbaugh PJ (2009). A core gut microbiome in obese and lean twins. Nature.

[34] Mariat D (2009). The firmicutes/bacteroidetes ratio of the human microbiota changes with age. BMC Microbiol..

[35] Hulan HW, Ackman RG, Ratnayake WM, Proudfoot FG (1989). Omega-3 fatty acid levels and general performance of commercial broilers fed practical levels of redfish meal. Poult. Sci..

[36] Poorghasemi M, Seidavi A, Qotbi AAA, Laudadio V, Tufarelli V (2013). Influence of dietary fat source on growth performance responses and carcass traits of broiler chicks. Asian Austral. J. Anim..

[37] Laudadio V, Tufarelli V (2011). Dehulled‐micronised lupin (lupinus albus l. cv. multitalia) as the main protein source for broilers: influence on growth performance, carcass traits and meat fatty acid composition. J. Sci. Food Agric..

[38] Howe P, Meyer B, Record S, Baghurst K (2006). Dietary intake of long-chain omega-3 polyunsaturated fatty acids: contribution of meat sources. Nutrition.

[39] Stahl LA, Begg DP, Weisinger RS, Sinclair AJ (2008). The role of omega-3 fatty acids in mood disorders. Curr. Opin. Investig. Drugs.

[40] Ciccone MM (2013). The role of omega-3 polyunsaturated fatty acids supplementation in childhood: a review. Recent Pat. Cardiovasc. Drug Discov..

[41] Ford Alexander C, Quigley Eamonn M M, Lacy Brian E, Lembo Anthony J, Saito Yuri A, Schiller Lawrence R, Soffer Edy E, Spiegel Brennan M R, Moayyedi Paul (2014). Efficacy of Prebiotics, Probiotics, and Synbiotics in Irritable Bowel Syndrome and Chronic Idiopathic Constipation: Systematic Review and Meta-analysis. American Journal of Gastroenterology.

[42] Ruggiero P (2014). Use of probiotics in the fight against Helicobacter pylori. WJGP..

[43] Wang ZK, Yang YS, Stefka AT, Sun G, Peng LH (2014). Review article: fungal microbiota and digestive diseases. Aliment. Pharmacol. Ther..

[44] Erdogan A, Rao SS (2015). Small intestinal fungal overgrowth. Curr. Gastroenterol Rep..

[45] Wang H (2017). Controlling of growth performance, lipid deposits and fatty acid composition of chicken meat through a probiotic, lactobacillus johnsonii, during subclinical clostridium perfringens, infection. Lipids Health Dis..

[46] Hossain ME, Kim GM, Lee SK, Yang CJ (2012). Growth performance, meat yield, oxidative stability, and fatty acid composition of meat from broilers fed diets supplemented with a medicinal plant and probiotics. Asian. Austral. J. Anim..

[47] Shin NR (2013). An increase in the akkermansia spp. population induced by metformin treatment improves glucose homeostasis in diet-induced obese mice. Gut.

[48] Everard A, Belzer C, Geurts L, Ouwerkerk JP, Cani PD (2013). Cross-talk between akkermansia muciniphila and intestinal epithelium controls diet-induced obesity. Proc. Natl. Acad. Sci. USA.

[49] Madigan, M. & Martinko, J. eds Brock Biology of Microorganisms (11th ed.). Prentice Hall, Upper Saddle River, NJ, USA. (2005).

[50] Sukhija P, Palmquist D (1988). Rapid method for determination of total fatty acid content and composition of feedstuffs and feces. J. Agr. Food Chem..

[51] Elkin R (2009). Additional perspectives on analytical techniques and standardization: Cholesterol and fatty acid contents of eggs, tissues, and organs. Poult. Sci..

[52] Edgar RC (2013). Uparse: highly accurate otu sequences from microbial amplicon reads. Nat. Methods.

[53] Zhang X (2015). Illumina miseq sequencing reveals diverse microbial communities of activated sludge systems stimulated by different aromatics for indigo biosynthesis from indole. Plos One.

[54] Cole James R., Wang Qiong, Fish Jordan A., Chai Benli, McGarrell Donna M., Sun Yanni, Brown C. Titus, Porras-Alfaro Andrea, Kuske Cheryl R., Tiedje James M. (2013). Ribosomal Database Project: data and tools for high throughput rRNA analysis. Nucleic Acids Research.

[55] Wang Q, Garrity GM, Tiedje JM, Cole JR (2007). Naive bayesian classifier for rapid assignment of rrna sequences into the new bacterial taxonomy. Appl. Environ. Microb..

[56] Caporaso J Gregory, Kuczynski Justin, Stombaugh Jesse, Bittinger Kyle, Bushman Frederic D, Costello Elizabeth K, Fierer Noah, Peña Antonio Gonzalez, Goodrich Julia K, Gordon Jeffrey I, Huttley Gavin A, Kelley Scott T, Knights Dan, Koenig Jeremy E, Ley Ruth E, Lozupone Catherine A, McDonald Daniel, Muegge Brian D, Pirrung Meg, Reeder Jens, Sevinsky Joel R, Turnbaugh Peter J, Walters William A, Widmann Jeremy, Yatsunenko Tanya, Zaneveld Jesse, Knight Rob (2010). QIIME allows analysis of high-throughput community sequencing data. Nature Methods.

[57] Kemp PF, Aller JY (2010). Bacterial diversity in aquatic and other environments: what 16s rdna libraries can tell us. FEMS Microbiol. Ecol..

[58] Yu Z, Yang J, Liu L, Zhang W, Amalfitano S (2015). Bacterioplankton community shifts associated with epipelagic and mesopelagic waters in the southern ocean. Sci. Rep..

[59] Sanders, J. G. *et al*. Baleen whales host a unique gut microbiome with similarities to both carnivores and herbivores. *Nat*. *Commun*. 6(8285), 10.1038/ncomms9285 (2015). 10.1038/ncomms9285 PMC4595633 26393325

[60] Segata N (2011). Metagenomic biomarker discovery and explanation. Genome Biol..

[61] Langille MG (2013). Predictive functional profiling of microbial communities using 16s rrna marker gene sequences. Nat. Biotechnol..

[62] Javurek AB (2016). Discovery of a novel seminal fluid microbiome and influence of estrogen receptor alpha genetic status. Sci. Rep..

[63] Mitteer Daniel R., Greer Brian D., Fisher Wayne W., Cohrs Victoria L. (2018). Teaching behavior technicians to create publication-quality, single-case design graphs in graphpad prism 7. Journal of Applied Behavior Analysis.

