# Supplementary material for: Correlation between Jejunal Microbial Diversity and Muscle Fatty Acids Deposition in Broilers Reared at Different Ambient Temperatures
Source: Sci Rep. 2019 Jul 30;9:11022. doi: 10.1038/s41598-019-47323-0 (PMC6667446; doi:10.1038/s41598-019-47323-0)
Supplement: Supplementary file 1 — Supplementary Material [file 41598_2019_47323_MOESM1_ESM.doc]

# Supplementary Information

# Correlation between Jejunal Microbial Diversity and Muscle Fatty Acids Deposition in Broilers Reared at Different Ambient Temperatures

Xing Li^1#^, Zhenhui Cao^1#^, Yuting Yang^1#^, Liang Chen^2^, Jianping Liu^3^, Qiuye Lin^4^, Yingying Qiao^1^, Zhiyong Zhao^5^, Qingcong An^1^, Chunyong Zhang^1^, Qihua Li^1^, Qiaoping Ji^6^, Hongfu Zhang^2*^, Hongbin Pan^1*^

^1^Yunnan Provincial Key Laboratory of Animal Nutrition and Feed Science, Faculty of Animal Science and Technology, Yunnan Agricultural University, Kunming 650201 China;

^2^State Key Laboratory of Animal Nutrition, Institute of Animal Science, Chinese Academy of Agricultural Sciences, Beijing100193, China;

^3^Jiangsu Key Laboratory for Molecular and Medical Biotechnology, College of Life Sciences, Nanjing Normal University, Nanjing 210023, China;

^4^College of Food Science and Technology, Yunnan Agricultural University, Kunming 650201, China;

^5^Yunnan Animal Science and Veterinary Institute, Kunming 650201, China;

^6^Yunnan Mudao Biology Technology Co., Ltd., Kunming 650201, China.

^#^These authors contribute equally to this work.

^*^Correspondence: Hongbin Pan, [ynsdyz@163.com](mailto:ynsdyz@163.com); Hongfu Zhang, zhanghongfu@caas.cn

**Table S1. Temperature management**^1^**.**

| Day | HJ group | MJ group | LJ group |
| --- | --- | --- | --- |
| 1 | 36.5 | 33.5 | 30.5 |
| 2 | 36 | 33 | 30 |
| 3 | 35.5 | 32.5 | 29.5 |
| 4 | 35 | 32 | 29 |
| 5 | 34.5 | 31.5 | 28.5 |
| 6 | 34 | 31 | 28 |
| 7 | 33.5 | 30.5 | 27.5 |
| 8 | 33 | 30 | 27 |
| 9 | 32.5 | 29.5 | 26.5 |
| 10 | 32 | 29 | 26 |
| 11 | 32 | 29 | 26 |
| 12 | 31.5 | 28.5 | 25.5 |
| 13 | 31 | 28 | 25 |
| 14 | 31 | 28 | 25 |
| 15 | 30.5 | 27.5 | 24.5 |
| 16 | 30.5 | 27.5 | 24.5 |
| 17 | 30 | 27 | 24 |
| 18 | 30 | 27 | 24 |
| 19 | 29.5 | 26.5 | 24.5 |
| 20 | 29 | 26 | 23 |
| 21 | 29 | 26 | 23 |
| 22 | 28.5 | 25.5 | 22.5 |
| 23 | 28 | 25 | 22 |
| 24 | 28 | 25 | 22 |
| 25 | 27.5 | 24.5 | 21.5 |
| 26 | 27.5 | 24.5 | 21.5 |
| 27 | 27 | 24 | 21 |
| 28 | 27 | 24 | 21 |
| 29 | 26.5 | 23.5 | 20.5 |
| 30 | 26 | 23 | 20 |
| 31 | 26 | 23 | 20 |
| 32 | 25.5 | 22.5 | 19.5 |
| 33 | 25 | 22 | 19 |
| 34 | 25 | 22 | 19 |
| 35 | 24.5 | 21.5 | 18.5 |
| 36 | 24 | 21 | 18 |
| 37 | 24 | 21 | 18 |
| 38 | 23.5 | 20.5 | 17.5 |
| 39 | 23 | 20 | 17 |
| 40 | 23 | 20 | 17 |
| 41 | 22.5 | 19.5 | 16.5 |
| 42 | 22 | 19 | 16 |

^1^When the temperature is 0.5 degree lower than the expected temperature, the heater is turned on and the fan is closed. When the heater is 0.5 degree higher, the heater is closed and the ventilator opens.

**Table S2.** Ingredient and nutrient composition (%, as feed) of the experimental diets.

| Ingredient (%) | Starter diet (%) 1 to 21d | Finisher diet (%) 22 to 42 d |
| --- | --- | --- |
| Corn, first | 60.00 | 66.40 |
| Soybean meal 43 | 27.40 | 20.00 |
| corn protein flour | 6.00 | 8.00 |
| Imported fish meal | 1.00 | 0.00 |
| Soybean oil | 1.90 | 1.60 |
| Stone powder | 1.10 | 1.20 |
| Calcium hydrogen phosphate | 1.10 | 1.14 |
| salt | 0.35 | 0.35 |
| Methionine | 0.07 | 0.06 |
| lysine | 0.08 | 0.25 |
| 1% broiler premix | 1.00 | 1.00 |
| Calculated nutrients level, % | | |
| ME, Mcal/kg | 2950 | 3000 |
| Crude protein | 21.00 | 19.00 |
| Calcium | 0.81 | 0.80 |
| Phosphorus | 0.50 | 0.50 |
| lysine | 1.10 | 1.00 |
| Methionine | 0.50 | 0.38 |
| Methionine + Cystine | 0.90 | 0.72 |
| Threonine | 0.80 | 0.74 |
| Tryptophan | 0.20 | 0.18 |

^1^ME: Metabolic energy.

**Table S3.** Description of the assembly results of jejunum microbiota from chicken Broiler.

| Sample name | Clean Reads | Mapped reads | Mapped ratio (%) | Bases(bp) | Q20 (%) | GC (%) | Average length(bp) | OTUs |
| --- | --- | --- | --- | --- | --- | --- | --- | --- |
| HJ-1 | 35237 | 33992 | 96.46678 | 15043100 | 94.66 | 51.54 | 426 | 204 |
| HJ-2 | 55762 | 51434 | 92.23844 | 23693421 | 93.09 | 52.56 | 424 | 366 |
| HJ-3 | 59964 | 58208 | 97.07158 | 25287490 | 95.38 | 53.06 | 421 | 399 |
| HJ-4 | 58524 | 56943 | 97.29854 | 24973267 | 94.82 | 51.35 | 426 | 238 |
| HJ-5 | 58624 | 57832 | 98.64902 | 25031311 | 95.30 | 50.92 | 426 | 135 |
| HJ-6 | 60057 | 58803 | 97.91198 | 25476213 | 95.40 | 50.72 | 424 | 206 |
| HJ-7 | 57801 | 56361 | 97.50869 | 24178169 | 95.50 | 51.26 | 418 | 336 |
| HJ-8 | 55392 | 52581 | 94.92526 | 23515672 | 95.36 | 51.43 | 424 | 146 |
| HJ-9 | 64441 | 63549 | 98.61579 | 27552970 | 95.14 | 51.00 | 427 | 100 |
| HJ-10 | 58944 | 57984 | 98.37134 | 25169141 | 95.53 | 51.26 | 427 | 194 |
| HJ-11 | 59426 | 57903 | 97.43715 | 25423721 | 94.93 | 51.29 | 427 | 140 |
| HJ-12 | 47446 | 46096 | 97.15466 | 20295813 | 94.93 | 51.38 | 427 | 220 |
| MJ-1 | 63921 | 60382 | 94.46348 | 27274259 | 95.09 | 51.43 | 426 | 174 |
| MJ-2 | 42438 | 40858 | 96.27692 | 17897158 | 94.62 | 53.40 | 421 | 185 |
| MJ-3 | 62621 | 57980 | 92.58875 | 26488566 | 94.20 | 52.38 | 422 | 342 |
| MJ-4 | 56308 | 54448 | 96.69674 | 23858570 | 95.15 | 53.37 | 423 | 201 |
| MJ-5 | 52912 | 51258 | 96.87406 | 22525692 | 94.46 | 53.54 | 425 | 114 |
| MJ-6 | 64234 | 62056 | 96.60927 | 27498649 | 94.89 | 51.29 | 428 | 142 |
| MJ-7 | 55451 | 52848 | 95.30577 | 23313045 | 95.37 | 53.16 | 420 | 240 |
| MJ-8 | 57638 | 55727 | 96.68448 | 24502566 | 95.06 | 53.49 | 425 | 145 |
| MJ-9 | 52089 | 50095 | 96.17194 | 22135420 | 94.21 | 53.59 | 424 | 134 |
| MJ-10 | 51841 | 49922 | 96.2983 | 22100170 | 94.32 | 54.23 | 426 | 90 |
| MJ-11 | 59338 | 56792 | 95.70933 | 24428301 | 96.04 | 52.92 | 411 | 126 |
| MJ-12 | 58079 | 56566 | 97.39493 | 24702743 | 95.24 | 53.45 | 425 | 110 |
| LJ-1 | 59692 | 57283 | 95.96428 | 24692711 | 95.65 | 52.0 | 413 | 296 |
| LJ-2 | 59834 | 56907 | 95.10813 | 25280142 | 95.20 | 53.10 | 422 | 303 |
| LJ-3 | 49053 | 47663 | 97.16633 | 20952159 | 94.90 | 51.42 | 427 | 275 |
| LJ-4 | 53172 | 50896 | 95.71955 | 22550040 | 95.08 | 54.19 | 424 | 117 |
| LJ-5 | 51757 | 49400 | 95.44603 | 21794950 | 94.86 | 53.67 | 421 | 234 |
| LJ-6 | 37678 | 35542 | 94.33091 | 16021863 | 94.63 | 50.80 | 425 | 198 |
| LJ-7 | 59759 | 57261 | 95.81988 | 25530205 | 93.48 | 52.06 | 427 | 244 |
| LJ-8 | 59218 | 56252 | 94.99139 | 24927752 | 95.36 | 52.90 | 420 | 337 |
| LJ-9 | 56192 | 53961 | 96.02968 | 22991523 | 95.99 | 52.27 | 409 | 255 |
| LJ-10 | 54886 | 52153 | 95.02059 | 23261150 | 95.06 | 53.60 | 423 | 227 |
| LJ-11 | 54469 | 47216 | 86.68417 | 22478354 | 95.40 | 55.83 | 412 | 230 |
| LJ-12 | 42064 | 36259 | 86.1996 | 17502712 | 92.80 | 53.60 | 416 | 361 |

**Table S4.** Diversity estimation of the 16S rRNA gene libraries of the chicken jejunum.

| Alpha name | OTUs | chao1 | Observed species | PD whole tree | shannon | simpson | Goods coverage |
| --- | --- | --- | --- | --- | --- | --- | --- |
| HJ-1 | 204 | 210.37 | 201 | 18.0530 | 3.3557 | 0.8036 | 0.9992 |
| HJ-2 | 366 | 390.56 | 363 | 28.8652 | 4.7243 | 0.8922 | 0.9982 |
| HJ-3 | 399 | 466.37 | 391 | 25.9980 | 4.0268 | 0.8120 | 0.9966 |
| HJ-4 | 238 | 252.88 | 235 | 20.7855 | 3.1733 | 0.7751 | 0.9989 |
| HJ-5 | 135 | 171.15 | 133 | 12.3537 | 1.3891 | 0.4184 | 0.9989 |
| HJ-6 | 206 | 276.70 | 203 | 17.8009 | 2.5838 | 0.6766 | 0.9977 |
| HJ-7 | 336 | 388.60 | 331 | 22.9336 | 5.1744 | 0.9295 | 0.9978 |
| HJ-8 | 146 | 216.72 | 145 | 13.8416 | 1.8219 | 0.5265 | 0.9978 |
| HJ-9 | 100 | 103.55 | 95 | 9.8902 | 0.7635 | 0.2036 | 0.9993 |
| HJ-10 | 194 | 217.33 | 189 | 16.1050 | 1.0528 | 0.2243 | 0.9983 |
| HJ-11 | 140 | 148.15 | 136 | 13.1100 | 2.5888 | 0.7360 | 0.9992 |
| HJ-12 | 220 | 291.02 | 216 | 20.3237 | 2.4626 | 0.7199 | 0.9974 |
| MJ-1 | 174 | 221.33 | 170 | 15.4792 | 2.2173 | 0.5502 | 0.9981 |
| MJ-2 | 185 | 198.60 | 185 | 15.3048 | 3.9587 | 0.7747 | 0.9994 |
| MJ-3 | 342 | 389.46 | 341 | 26.1019 | 5.0170 | 0.8886 | 0.9988 |
| MJ-4 | 201 | 216.33 | 197 | 17.1816 | 2.7389 | 0.6891 | 0.9990 |
| MJ-5 | 114 | 121.42 | 112 | 10.8142 | 2.4893 | 0.6206 | 0.9996 |
| MJ-7 | 240 | 287.00 | 237 | 17.9707 | 5.1020 | 0.8787 | 0.9991 |
| MJ-8 | 145 | 157.60 | 144 | 15.4058 | 2.4927 | 0.6478 | 0.9994 |
| MJ-9 | 134 | 149.00 | 132 | 14.8966 | 2.7043 | 0.6028 | 0.9994 |
| MJ-10 | 90 | 100.00 | 87 | 10.5202 | 2.3619 | 0.6530 | 0.9995 |
| MJ-11 | 126 | 186.14 | 123 | 12.9235 | 1.7877 | 0.5571 | 0.9982 |
| MJ-12 | 110 | 118.42 | 109 | 12.8264 | 2.3814 | 0.6182 | 0.9996 |
| LJ-1 | 296 | 378.20 | 293 | 19.0362 | 4.6434 | 0.8833 | 0.9976 |
| LJ-2 | 303 | 336.55 | 296 | 23.0743 | 3.8151 | 0.7950 | 0.9983 |
| LJ-3 | 275 | 365.23 | 270 | 20.3857 | 2.6990 | 0.7347 | 0.9969 |
| LJ-4 | 117 | 123.20 | 116 | 11.0414 | 3.4838 | 0.7786 | 0.9997 |
| LJ-5 | 234 | 264.05 | 229 | 22.2638 | 3.4765 | 0.8246 | 0.9987 |
| LJ-6 | 198 | 211.50 | 195 | 19.6502 | 3.8315 | 0.8697 | 0.9992 |
| LJ-7 | 244 | 271.68 | 242 | 22.1308 | 3.4267 | 0.8453 | 0.9986 |
| LJ-8 | 337 | 358.37 | 335 | 26.6157 | 4.8401 | 0.8837 | 0.9988 |
| LJ-9 | 255 | 276.08 | 255 | 19.1435 | 5.6836 | 0.9600 | 0.9992 |
| LJ-10 | 227 | 263.05 | 222 | 18.8010 | 3.2021 | 0.6892 | 0.9986 |
| LJ-11 | 230 | 257.56 | 227 | 22.2577 | 5.0730 | 0.9495 | 0.9987 |
| LJ-12 | 361 | 416.27 | 359 | 27.7911 | 6.7991 | 0.9762 | 0.9988 |


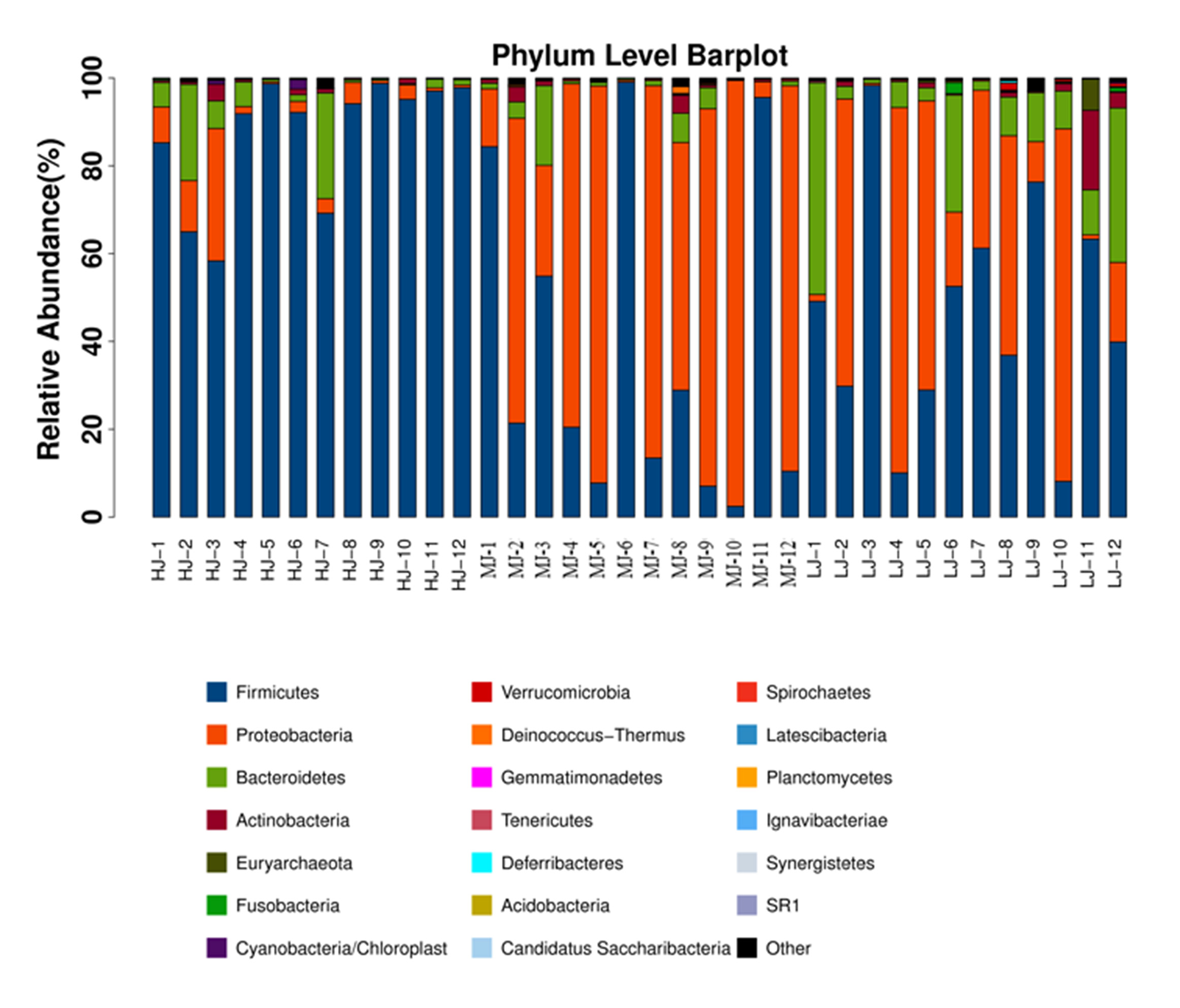


**Figure S1.** Taxonomy profile of microbiota composition at the phylum level.


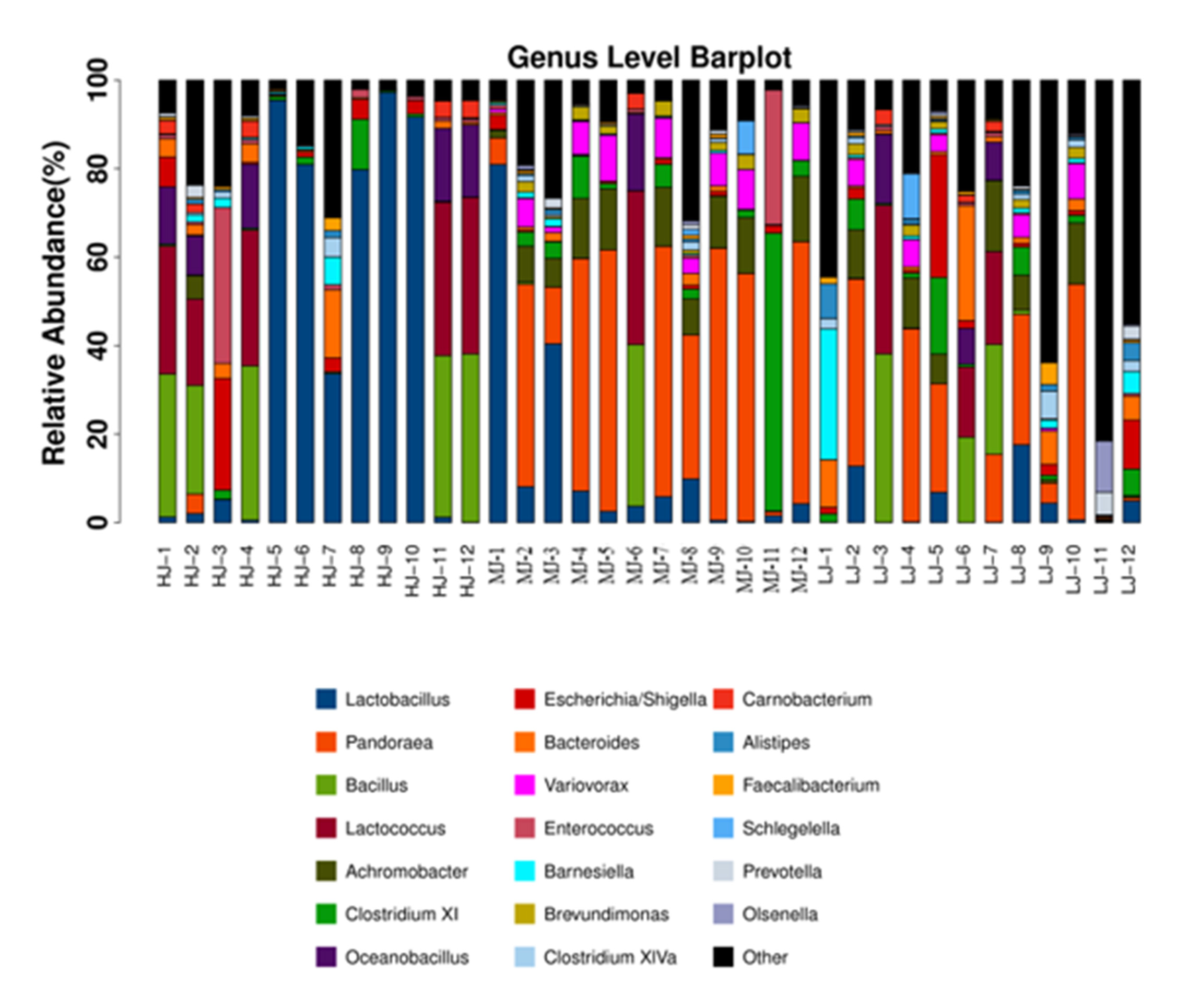


**Figure S2.** Taxonomy profile of microbiota composition at the genus level.


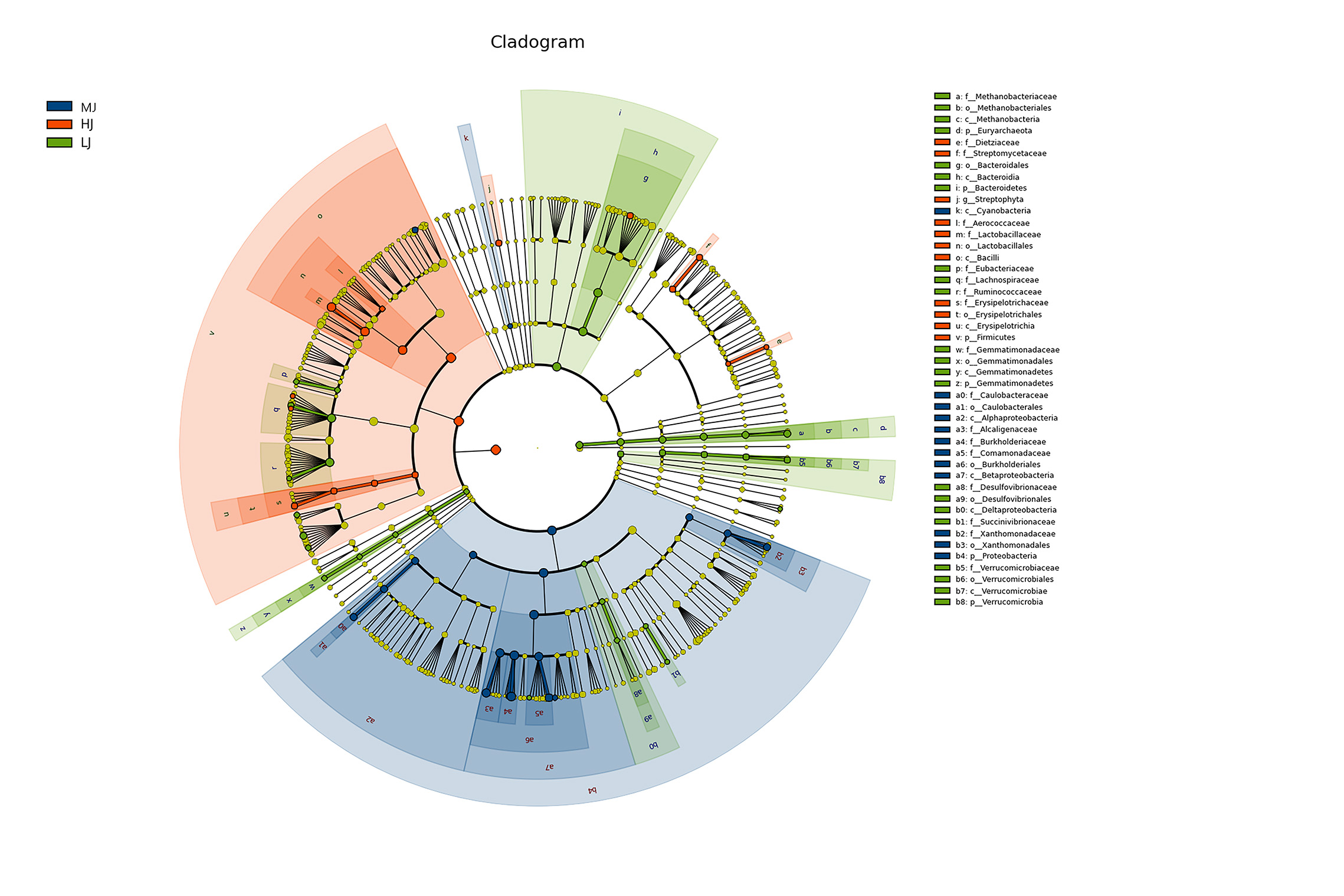


**Figure S3.** Species cladogram obtained from LEfSe analysis of 16 S rRNA sequences between HJ, MJ and LJ groups. (Red) HJ-enriched species, (Green) species enriched in LJ, (Blue) species enriched in MJ; the brightness of each dot is proportional to its effect size.
